# Walking for Health: Franz Tappeiner (1816–1902), Meran, and the Origins of Public Health-Oriented Physical Activity

**DOI:** 10.3390/ijerph23020248

**Published:** 2026-02-16

**Authors:** Christian J. Wiedermann, Patrick Rina, Ulrike Kindl, Doris Hager von Strobele Prainsack

**Affiliations:** 1 Institute of General Practice and Public Health, Claudiana College of Health Professions, 39100 Bolzano, Italy; 2 Department of Linguistics and Comparative Cultural Studies, Ca’ Foscari University, 30123 Venice, Italy

**Keywords:** Franz Tappeiner, Terrainkur, rehabilitation, climate-sensitive medicine, green infrastructure, Meran, health citizenship, urban public health, walkability, preventive medicine

## Abstract

**Highlights:**

**Public health relevance—How does this work relate to a public health issue?**

- Examines early use of walking and environmental design as population-level strategies to promote physical activity and prevent chronic disease.
- Links 19th-century terrain-based walking in Meran to contemporary public health challenges, including inactivity and urban health inequities.

**Public health significance—Why is this work of significance to public health?**

- Demonstrates that structured, accessible walking environments were implemented as public health interventions well before modern exercise guidelines.
- Provides historical evidence that physical activity promotion can be embedded in civic infrastructure rather than relying solely on individual behavior change.

**Public health implications—What are the key implications or messages for practitioners, policy makers and/or researchers in public health?**

- Supports walkable green infrastructure as a scalable, low-cost public health intervention for prevention and rehabilitation across populations.
- Encourages integration of physical activity, urban planning, and environmental health in public health research and policy development.

**Abstract:**

Background/Objectives: Franz Tappeiner (1816–1902) is often celebrated as a pioneer of alpine medicine and the founder of Tappeiner Promenade in Meran (South Tyrol, Italy). However, his legacy extends far beyond the scenic infrastructure, encompassing a comprehensive vision of physical activity as a public health intervention. His multidisciplinary practice anticipated the principles of contemporary rehabilitation, preventive medicine, and climate-sensitive public health. Methods: This historical public health analysis, combining biographical, contextual, and material–spatial approaches, reinterprets Tappeiner’s writings, institutional engagements, and civic projects through the lens of modern public health frameworks. Drawing on primary materials (e.g., published articles, autobiographical fragments, and commemorative texts) and recent evidence from rehabilitation and environmental health research, these contributions were contextualized. Results: Tappeiner’s early focus on infectious disease prevention (e.g., cholera and tuberculosis) transitioned into a strategic emphasis on recovery and behavioral therapy through environmental design. The walking therapy model of Max Joseph Oertel, locally realized in the Tappeiner Promenade, prefigured modern concepts such as structured green rehabilitation, walkability, and urban-health citizenship. His systematic integration of graded walking into civic infrastructure represents one of the earliest documented examples of embedding physical activity promotion at the population level. He contributed substantial personal funds to the path’s construction, embedding therapeutic gradients, curating vegetation, and promoting inclusive design to support convalescence. Contemporary research supports the intuition that green, low- to moderate-intensity walking improves cardiometabolic health, psychological well-being, and functional capacity. Moreover, his integrative ethos, merging clinical medicine, civic ethics, and spatial intervention, parallels contemporary eco-social models of public health. Conclusions: Franz Tappeiner’s career exemplifies a still-relevant model of physician leadership that is empirically grounded, socially accountable, and ecologically attuned, with physical activity promotion embedded as a central element of his public health vision. His work invites reflection on how medical professionals can shape not only individual care but also urban environments and collective health futures.

## 1. Introduction

Dr. Franz Tappeiner (1816–1902) is recognized as a central figure in the emergence of Meran (German name; Italian: Merano) as a 19th-century alpine health resort [1]. Throughout this article, the historical name Meran is used, reflecting the official German place name during Franz Tappeiner’s lifetime. Tappeiner championed the integration of climatic and landscape-based walking therapy (*Terrainkur*), a model popularized by the Munich laryngologist Max Joseph Oertel [2], alongside social hygiene, epidemic control, and civic health planning [3,4]. Central to his approach was the conviction that structured physical activity—particularly walking in therapeutically designed environments—constituted a fundamental element of disease prevention and health recovery.

Its relevance has resurfaced considering modern challenges, such as chronic disease prevention through physical activity, climate-responsive healthcare, and nature-based rehabilitation strategies, which increasingly echo historical practices once sidelined by biomedical reductionism [5,6]. His work during the 1855 cholera epidemic in Meran exemplifies early epidemiological communication and hygiene education, and his financing of the *Terrainkur* walkway “Tappeiner Promenade” (Tappeinerweg) illustrates the concretization of nature as a therapeutic setting [1,7].

This article is published in conjunction with the commemoration of Tappeiner’s 210th birthday in 2026, which is being marked by a matinée event at Meran’s city library on 17 January 2026. Bringing together voices from cultural history, journalism, and medicine, the event frames Tappeiner as both a product and shaper of 19th-century modernity.

Drawing from both primary material (including educational records and local health policy initiatives) and secondary medical-historical research, this article reconstructs Tappeiner’s development as a physician and a civic actor. A historiographically informed perspective situates him within the epistemic landscape of medicine around 1850, a period marked by theoretical pluralism, diagnostic uncertainty, and competing paradigms of disease causation.

The goals of this study are threefold:

- To contextualize Tappeiner’s medical and civic activity within 19th-century Central European health culture;
- To evaluate his contributions considering modern public health discourse—particularly regarding physical activity promotion, environmental and preventive care;
- To assess the continued relevance of terrain-based and climate-responsive health models in rehabilitation and noncommunicable disease management today.

Rather than offering a chronological biography, this article proceeds thematically. It begins with a brief biographical sketch and historical context, proceeds to analyze prevailing medical theories and practices around 1850, and then turns to Tappeiner’s applied work in Meran, especially his roles in epidemic response, health education, and spatial-therapeutic innovation. The conclusion reflects on his enduring legacy in medical humanism and public health practice.

### Methodological Approach: Historical Public Health Analysis

This study combines biographical historical research, contextual analysis, and material–spatial analysis. Primary sources include Franz Tappeiner’s published medical writings, pamphlets, experimental reports, contemporaneous newspaper articles, and archival materials on Meran’s medical, hygienic, and urban development. We interpreted these sources within their 19th-century context, considering prevailing medical theories (miasmatic and early contagionist models) and public health practices.

Secondary sources from medical history, public health history, and environmental health research contextualize Tappeiner’s work and assess its relevance for contemporary public health frameworks: preventive medicine, rehabilitation, and urban health.

We analyzed the Tappeiner Promenade as health-related civic infrastructure, examining its design features, accessibility, gradients, and intended therapeutic use as documented in historical sources.

The analysis is interpretative, not quantitative. It situates Tappeiner’s medical and civic practices within public health thinking and links them to current evidence on physical activity, green infrastructure, and climate-sensitive health promotion.

## 2. Biographical Sketch and Historical Context

### 2.1. Rural Origins and Academic Formation

Franz Tappeiner was born in 1816 in Laas (Lasa), a rural village in the Vinschgau valley of the historic Tyrol, today South Tyrol (Autonomous Province of Bolzano, Italy). Coming from a farming family, his path to the medical profession was marked by intellectual ambition and a remarkable educational trajectory for someone of a non-aristocratic background. After attending the Benedictine Gymnasium in Meran and completing his gymnasium and philosophical studies in Innsbruck, Tappeiner began his medical studies in 1836 at the University of Prague. He continued his medical education in Padua in 1837 and subsequently returned to Prague, before completing his studies in Vienna, where he came under the influence of leading figures of the Second Viennese Medical School. He attended clinical and pathological lectures by Joseph Škoda and Carl von Rokitansky, and dermatology lectures by Ferdinand von Hebra. These formative experiences combined the traditional framework of humoral theory with the emerging empirical-scientific approach to diagnosis and pathology. He was awarded a Doctor medicinae universae degree in 1843 [1,8].

**Figure 1:**
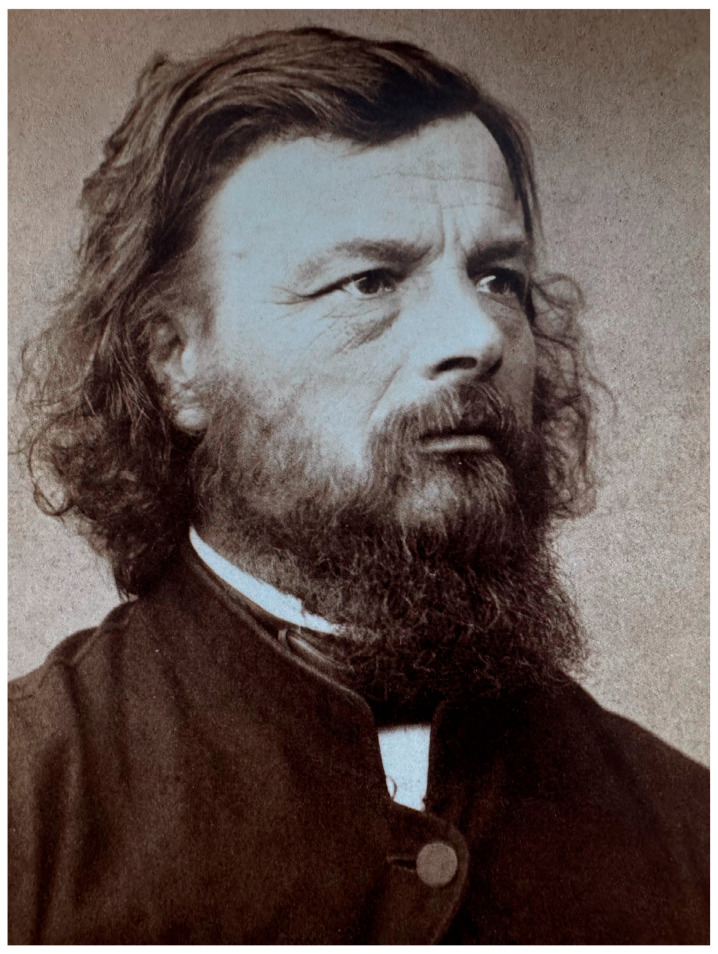
Portrait of Dr. Franz Tappeiner (around 1870). Image courtesy of the Palais Mamming Museum, Meran. © Palais Mamming Museum Meran.

In 1846, after completing his medical studies and initial clinical experience, Tappeiner moved to Meran to establish himself as a practicing physician in a town marked by post-revolutionary uncertainty, poverty, and inadequate public health infrastructure (Figure 1). At that time, Meran lacked a proper hospital and was served only by a basic hospice and an old-age care facility. With few medical professionals and recurring epidemics worsened by inadequate sanitation, these conditions strengthened Tappeiner’s enduring dedication to public hygiene, modern medical infrastructure, and community-based health initiatives [1,8].

### 2.2. Political and Social Conditions in 19th-Century Tyrol

The period following the Napoleonic Wars saw Tyrol reintegrated into the Austrian Empire, experiencing both administrative modernization and growing nationalist sentiment [9,10]. The Vormärz era was marked by socio-political rigidity, as well as infrastructural and intellectual stirrings [11]. In South Tyrol, the economy remained predominantly agrarian throughout the first half of the 19th century, with small-scale handicrafts often being integrated into agricultural households. However, by the late 1830s and the 1840s, urban centers such as Meran began to develop a civic bourgeoisie that was increasingly invested in public hygiene, education, and emerging medical modernity, particularly as the town gained recognition for its health-promoting climate and therapeutic potential [1,7,12].

### 2.3. Medical Theory and Practice Circa 1850

Tappeiner practiced at a time when the epistemic foundations of medicine were in a state of flux. Germ theory had not yet been established—Koch’s postulates emerged only in the 1880s—and miasma theory, which attributed disease to harmful environmental vapors or “bad air,” remained dominant in European medical thinking [13,14]. Physicians believed that clean air, proper elevation, and natural environments were essential for preventing and curing illnesses, particularly respiratory and epidemic diseases [15,16]. This context explains Tappeiner’s therapeutic emphasis on mountain climates, fresh air, and atmospheric purity, aligning him with the broader 19th-century practices of climate therapy and high-altitude health resorts [17,18].

Contagionism often coexisted with miasmatic theory, with physicians viewing both “bad air” and direct transmission as pathogenic forces requiring ventilation and isolation [19,20]. “Heroic” treatments, such as bloodletting and purgatives, remained common based on humoral models [21,22], while balneological treatments reflected environmental healing beliefs [23]. Pre-bacteriological hygiene entered institutions through hygiene chairs and sanitation protocols [24,25]. Limited pharmacological options have led to empirical yet theoretically speculative medicine [26,27].

Tappeiner’s adoption of terrain therapy, climate medicine, and public sanitation initiatives reveals a practitioner who was highly responsive to evolving conceptions of health. Rather than adhering rigidly to dogma, Tappeiner integrated empirical reasoning with a strong belief in environmental and social determinants, positioning himself at the forefront of mid-19th-century medicine. In this context, his later advocacy for structured walking as therapeutic intervention can be understood not as a peripheral activity but as a logical extension of his environmentally oriented medical reasoning.

## 3. Public Health Physician: Cholera, Hygiene, and Preventive Thinking

When Franz Tappeiner moved to Meran in 1846, he swiftly became a pivotal figure in the city’s medical and public spheres, influencing them for several years [1]. His medical practice extended beyond the city limits, reaching rural communities in the Vinschgau (Venosta) and Passeier (Passiria) valleys. In an area lacking adequate sanitary facilities, hospitals, and sufficient medical staff, he combined his clinical duties with public efforts to enhance hygiene, prevent diseases, and promote public health. Tappeiner exemplified the physician-citizen model by merging practical medical care with a commitment to environmental and social concerns.

### 3.1. The 1855 Cholera Pamphlet: Community-Facing Medical Communication

Cholera posed a recurring threat to 19th-century Northern Italy, with severe outbreaks occurring in 1836 and 1855. In 1855, nearly 30,000 people died in Tuscany alone [28], and Ferrara recorded over 2000 cases linked to poor housing conditions [29]. In Bologna, the epidemic triggered a shift from clerical to medical administrative governance [30]. Although data on South Tyrol are limited, the region lies within a vulnerable Alpine-Habsburg corridor. Nearby Carinthia and Carniola responded with quarantine regimes and lazarettos, reflecting the evolving epidemic governance [31,32,33].

In response to the devastating cholera epidemic of 1855 [28,29,30], Tappeiner published a leaflet titled “Some Words About Cholera for the Instruction and Reassurance of the Rural Population,” one of the rare examples of early medical outreach aimed directly at lay audiences in South Tyrol (Supplementary Figure S1 and File S1) [34]. The text was written during a time of widespread panic and misinformation, and it sought to calm fears and provide practical advice to the rural population, which he considered especially vulnerable to rumors and superstition.

Written for a lay audience, the pamphlet translated prevailing medical knowledge into practical behavioral guidance. Tappeiner urged readers to recognize mild, early diarrhea as the initial stage of cholera and stressed that timely action at this point was decisive. He recommended ventilation, personal hygiene, warmth, and moderation in diet, alongside simple, inexpensive remedies that could be administered without delay. Equally important, he cautioned against panic, extreme dietary restriction, and social withdrawal, arguing instead for calm conduct and assistance to affected individuals. By framing cholera as not contagious in general and emphasizing social solidarity, the pamphlet combined environmental hygiene with an early understanding of behavioral and psychosocial determinants of health.

### 3.2. Hygiene, Early Intervention, and Behavioral Regulation

As discussed in Section 2.3, miasmatic logic dominated mid-19th-century medicine. Within this framework, Tappeiner’s pamphlet advocated for prevailing medical paradigms. Contagionism emerged in hybrid models that linked disease spread to impure air [35,36]. Tappeiner’s emphasis on ventilation, cleanliness, and moderate eating aligns with contemporary preventive practices [37]. Although the pamphlet did not explicitly prescribe physical exercise, its emphasis on behavioral self-regulation, active health conduct, and maintaining bodily function during epidemics reflects a broader preventive logic that Tappeiner would later extend to structured physical activity as a population-level health strategy. His warnings against dietary excess and fear reflected humoral theories linking illness to imbalances and emotional disturbances [38,39,40]. This guidance embodied the “dietetics of the soul” and recognized emotions as pathogenic factors [39,40]. The pamphlet transformed the physicians into public health advocates. Through practical guidance and promotion of collective responsibility, Tappeiner pioneered epidemic risk communication [19,37], combining authority with humility while emphasizing practical health protection measures.

### 3.3. Assessment from a Contemporary Perspective

Tappeiner’s 1855 cholera pamphlet constitutes a notably progressive contribution to public health communication, even when evaluated using contemporary standards. Although grounded in the miasmatic paradigm, it articulates principles that are now integral to modern outbreak guidance: clear, actionable messaging, behavior-oriented prevention, culturally embedded language, and an early form of community health literacy [41,42,43]. His concise instructions on recognizing early symptoms, employing simple remedies, ensuring ventilation, and avoiding fear or excess reflected a pragmatic, experience-based approach to epidemic risk mitigation.

In contrast to abstract or medicalized explanations, Tappeiner’s approach emphasized practical behavioral modifications, such as sanitary discipline, dietary moderation, and psychological calmness, which align closely with contemporary evidence on effective cholera communication strategies [44,45]. Presented in local accessible prose, this approach exemplifies a model of culturally appropriate and community-specific messaging endorsed by modern risk communication frameworks [46,47].

Although the absence of microbial contagion may appear to be a deficit, it must be understood within the epistemic constraints of the 1850s. Tappeiner’s trust in environmental control and behavioral measures was both scientifically coherent for the period and strategically effective within its historical framework. His intervention thus reflects an early instance of prevention-first, population-level health promotion, foreshadowing today WASH (Water, Sanitation, and Hygiene) logic and One Health paradigms [45,48]. Furthermore, Tappeiner’s preventive orientation—centered on behavioral modification and environmental control rather than purely pharmacological intervention—laid the intellectual groundwork for his subsequent embrace of physical activity as a health-promoting strategy, linking epidemic hygiene to the later Terrainkur model.

## 4. The Researching Physician: Tappeiner Between Science and Practice

### 4.1. Scientific Inquiry Beyond Institutions

Despite being based outside the academic centers, Tappeiner undertook empirical investigations that reflected a rigorous scientific mindset. His meteorological recordings, climatological reports, and long-term observations of pulmonary patients exemplified an early form of translational research, turning local data into generalized health recommendations. His commitment to methodical, data-informed practice positions him within the lineage of “researching physicians” whose work bridges the bedside and benchside, clinic, and community.

### 4.2. Tuberculosis Transmission Experiments

Before the discovery of the tubercle bacillus in 1882, Franz Tappeiner had already positioned Meran as a favorable location for treating pulmonary diseases, especially phthisis (the historical term for what is now recognized as pulmonary tuberculosis), based on climatological and observational data. By the 1840s, Meran had begun attracting patients suffering from consumption (“Schwindsucht”), and Tappeiner’s publications played a central role in legitimizing its status as a therapeutic destination [49,50]. Although specialized sanatoria emerged only later, his advocacy for hygiene, outdoor exposure, and elevation helped lay the conceptual groundwork for Meran’s transformation into a tuberculosis health resort city. Notably, his direct involvement in climate medicine preceded his 1877 experimental work on tuberculosis transmission, reflecting a transition from empirical climatotherapy to pathogen-oriented investigation.

#### 4.2.1. Experimental Proof of Airborne Transmission

Beginning in 1877, Franz Tappeiner conducted experimental studies to clarify tuberculosis transmission pathways [51,52]. Using inhalation exposure to aerosolized sputum from tuberculosis patients, he demonstrated that phthisis could be transmitted via airborne particles, supporting inhalation rather than ingestion as the primary infection route [52,53,54,55]. These experiments were presented at the “Versammlung deutscher Naturforscher und Ärzte” in Munich and published in Virchow’s Archiv [56].

These findings preceded Robert Koch’s identification of the tubercle bacillus in 1882 and highlighted the role of air, ventilation, and environmental exposure in tuberculosis transmission before bacteriological proof existed [52,53,54,55]. Tappeiner’s work anticipated later public health concepts linking respiratory disease prevention to environmental control.

#### 4.2.2. Scientific Reception and Historical Positioning

Tappeiner’s experimental findings were discussed within contemporary medical discourse and cited in professional journals of the late 19th century [53,54,57]. His work was acknowledged by leading medical authorities, including Rudolf Virchow, with whom replication experiments were conducted in Berlin [55], Although controversial at the time and limited by the absence of microbiological techniques, these studies contributed to the evolving understanding of tuberculosis as an airborne disease and informed hygienic and preventive thinking prior to bacteriological confirmation.

#### 4.2.3. Enduring Relevance and Validation Through Modern Research

Microbiological and epidemiological research has confirmed the viability of Mycobacterium tuberculosis in aerosolized and dried sputum and the central role of ventilation and indoor air quality in transmission risk [58,59,60,61,62,63,64]. Studies demonstrate that improved ventilation and reduced crowding substantially lower airborne tuberculosis transmission in healthcare and community settings [59,60,61].

These findings support Tappeiner’s emphasis on fresh air, environmental control, and spatial conditions as preventive measures [51,54]. Though conducted without bacteriological tools, his experiments linked disease prevention to air quality and built environments, consistent with modern evidence on respiratory infection control. Moreover, the emphasis on outdoor air exposure and open environments as conditions for recovery anticipated the therapeutic rationale behind Terrainkur, in which physical activity in fresh air served simultaneously as a preventive and rehabilitative measure.

## 5. Public Health in Practice: From Hygiene to Therapeutic Landscapes

### 5.1. Hygienic Interventions and Preventive Thinking

Franz Tappeiner’s contributions to public health in Meran began with his proactive response to the cholera epidemic of 1855. His efforts extended beyond direct patient care and included public education initiatives. The pamphlet emphasized the importance of early symptom recognition, advocated straightforward behavioral and dietary interventions, and advised against panic. This approach was reflective of the prevailing miasmatic theory of disease while also highlighting the significance of behavioral prevention and emotional regulation.

Tappeiner’s methodology was founded upon the principles of education, environmental regulation, and social solidarity. He asserted that health should not be regarded as a private issue but as a matter of civic responsibility. Affluent citizens were encouraged to support the impoverished, medications were dispensed at no cost, and municipal authorities were urged to establish the essential infrastructure. His focus on clean water, well-ventilated housing, and systematic burial practices presaged numerous elements of institutional hygiene that emerged only in subsequent decades [1].

Importantly, Tappeiner’s approach to preventive health extended beyond addressing acute epidemics. He consistently perceived health as being influenced by modifiable factors such as air, water, and behavior, and acted in accordance with this understanding. He endorsed early quarantine measures, advocated for waste management reforms, and promoted public access to medical information. Through these initiatives, Meran established a foundational framework for a health-conscious city, predating the formalization of such concepts in public health legislation [1].

Tappeiner’s philosophy embodies an early iteration of what would subsequently be identified as “social hygiene”: a form of medicine intertwined with civic responsibility, moral conduct and environmental stewardship. His interventions established the foundation for Meran’s evolution from a provincial town into a therapeutic landscape characterized by health-oriented infrastructure rather than merely passive geography [1]. This transformation ultimately encompassed the systematic promotion of physical activity through publicly accessible walking infrastructure, establishing Meran as one of the earliest examples of a city designed to facilitate health-promoting movement at the population level.

### 5.2. Climate Medicine and the Rise of Meran as a Health Destination

**Figure 2:**
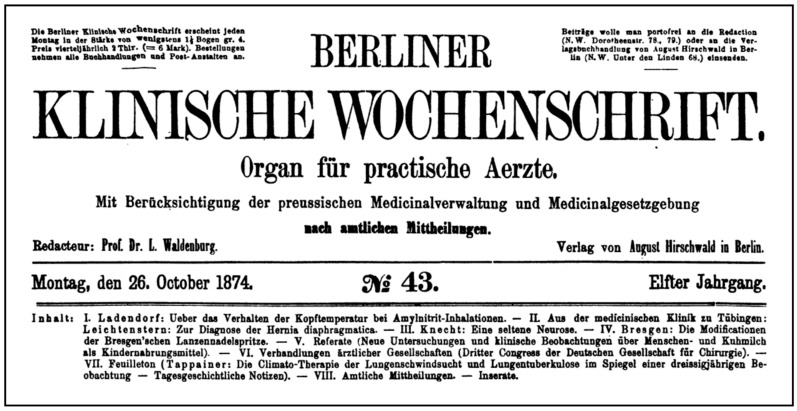
Tappeiner’s Climate Therapy in Medical Discourse: Feuilleton on Pulmonary Tuberculosis in the Berliner Klinische Wochenschrift, 26 October 1874 [50].

Prior to the establishment of the formal sanatorium system, Tappeiner played a pivotal role in establishing Meran as a site of therapeutic significance for individuals suffering from consumption [65], a term historically associated with phthisis and later identified as pulmonary tuberculosis [66]. Drawing from multi-year clinical observations and local meteorological data, he argued that Meran’s mild winters, moderate humidity, and stable barometric conditions created an optimal environment for respiratory recovery [49]. His reasoning, published in “*Die Climato-Therapie der Lungenkatharre und Lungenphthise im Spiegel einer dreißigjährigen Beobachtung*” (*The Climate Therapy of Pulmonary Catarrh and Pulmonary Phthisis in the Light of Thirty Years of Observation*) in 1874 [50], emphasized the role of empirical evidence gathered over decades of treating patients in Meran, rather than anecdotal reports or romanticized landscape depictions (Figure 2).

His argument followed a clinical, observation-based logic. Tappeiner documented seasonal patterns in disease progression and differentiated the outcomes of patients wintering in Meran from those treated in northern or alpine regions, where harsh conditions often triggered relapses or accelerated decline in health. He proposed that early relocation to a climate-stable environment could delay deterioration—an idea aligned with the constitutional thinking of the time but reinforced by longitudinal outcomes [50]. This climate-based rationale for pulmonary care played a key role in Meran’s rise as a therapeutic center before the bacteriological revolution in tuberculosis treatment.

Tappeiner’s contributions presaged the emergence of alpine sanatoria, such as those in Davos and Arosa, yet his methodology was conceptually distinct. He conceptualized climate and physical activity as modifiable determinants of health rather than merely passive geographic assets or individual leisure pursuits. Instead of relying on enclosed institutions, he integrated medical reasoning with civic infrastructure and advocated for the development of walkways, green spaces, and enhanced ventilation. His design of Meran as a therapeutic urban landscape anticipated the now widely accepted public health principle that cities can be engineered to promote good health.

This conceptual framework resonates significantly with contemporary research on Urban Green and Blue Spaces (UGBS) as health assets. Current studies confirm that meticulously designed urban greenways and park systems, such as the Tappeiner Promenade, promote physical activity, enhance air quality, mitigate urban heat, and support mental well-being, particularly for socioeconomically disadvantaged groups [67,68,69]. Furthermore, the present focus on integrating health objectives into urban planning, co-designing with citizens, and restoring ecological buffers aligns with the environmental foresight inherent in Tappeiner’s interventions [70,71,72]. His work anticipated what is now recognized as preventive urbanism, in which spatial design, green infrastructure, and health equity are interconnected. By reimagining Meran as a walkable, ventilated, and restorative environment, Tappeiner conceptualized the city as a living therapeutic system—an idea that is only now being formalized in urban health and climate policies.

### 5.3. The Terrainkur as Visionary Preventive Medicine

In the 1880s, Tappeiner began engaging with the *Terrainkur*, a physiologically grounded form of graded walking therapy systematically developed by von Oertel [73]. Alongside Tappeiner, physicians such as Bernhard Mazegger jun. and Raphael Hausmann played intermediary roles in operationalizing *Terrainkur* in Meran, linking Oertel’s physiological theory with local governance, alpine associations, and patient education [74,75]. Contemporary reporting in the Meraner Zeitung demonstrates that the introduction of the *Terrainkur* in Meran was not confined to medical circles but actively communicated to the wider public [75]. Detailed newspaper accounts explained its physiological rationale, therapeutic indications, and infrastructural realization, showing high public engagement with health, prevention, and climate-based therapy in late-19th-century Meran. This communication anticipated modern health promotion principles: accessible health information, empowerment through knowledge, and embedding preventive behaviors in everyday settings. These approaches align with contemporary frameworks emphasizing informed participation, supportive environments, and population-level engagement for sustainable behavior change.

The institutional involvement of the Alpine Club (Alpenverein) was pivotal for translating Oertel’s physiologically defined *Terrainkur* into a reproducible, scalable public health infrastructure. By designing, marking, and maintaining graded paths according to medical specifications, the Alpine Club functioned as an early mediator between clinical prescription and spatial implementation [74].

Rooted in cardiopulmonary physiology, *Terrainkur* was originally designed for patients with chronic lung disease, anemia, early cardiac decompensation, and what Oertel described as fatty heart disease—a metabolic form of myocardial dysfunction responsive to structured physical exertion [73]. Oertel’s approach emphasizes controlled ascents on defined gradients, with attention to pulse regulation, respiratory efficiency, and endurance building. The intervention aimed not only at somatic strengthening but also at optimizing oxygen uptake through prolonged exposure to open elevated air. Tappeiner, aware of the scientific underpinnings and therapeutic aims outlined by Oertel, strongly supported the integration of this regimen into the landscape of Meran, aligning it with his broader vision of preventive medicine and topography-based rehabilitation. In this regard, Tappeiner’s contribution transcended the mere endorsement of walking as a therapeutic modality; he recognized that physical activity, to achieve population-level health effects, required deliberate environmental design, public investment, and institutional coordination.

#### 5.3.1. Therapeutic Landscapes and Environmental Reasoning

The development of the *Terrainkur* walkway in the late 1880s marked a pivotal shift in Meran’s transformation into a modern health resort city. Sparked by the visit and influence of Oertel, the city embraced the principles of *Terrainkur* as walking therapy. At the request of the health resort administration, Oertel served as consulting physician between 1886 and 1892, guiding the implementation of graded walking paths, standardized route markings, and medically supervised exercise protocols [1,76]. Combining elevation-adjusted gradients, curated vegetation, and broad accessibility, the promenade embodied an open-air alternative to closed sanatoria. Its design reflected a civic and ecological vision of public health, grounded in the conviction that structured movement in fresh air, especially during Meran’s dry winter climate, could improve cardiopulmonary and metabolic function.

**Figure 3:**
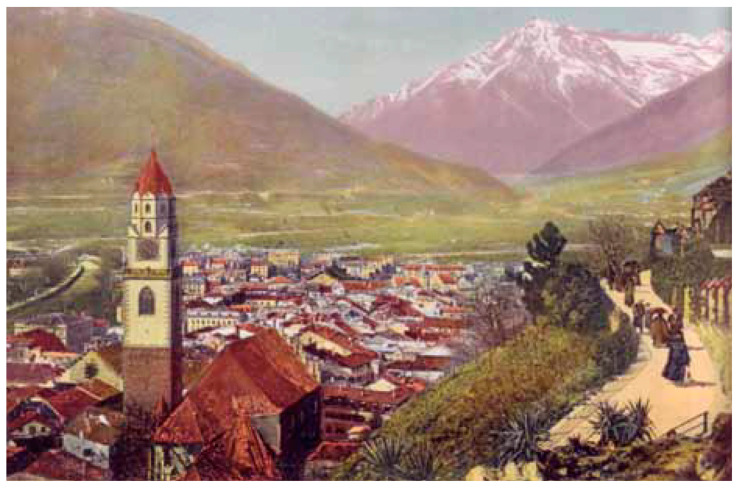
Historic colored postcard showing the Tappeiner Promenade with pedestrians on its landscaped path, overlooking the city of Meran and the prominent bell tower of the Parish Church. The scene illustrates the integration of therapeutic walking infrastructure with panoramic urban and alpine vistas. Image courtesy of the Palais Mamming Museum, Meran. © Palais Mamming Museum Meran.

Financed significantly through Tappeiner’s personal funds—amounting to 120,000 Kronen [1], which corresponds to approximately 700,000 Euros today—and developed in consultation with local horticulturists, the promenade included a path that accommodated users with different physical abilities (Figure 3). Its design reflects therapeutic logic: exposure to curated vegetation, elevation-adjusted movement, and panoramic views were intended to support vascular, pulmonary, and psychological health.

In contrast to enclosed, fee-based sanatoria that increasingly characterized tuberculosis treatment in alpine regions, the Tappeiner Promenade was freely accessible to all, irrespective of social class or diagnosis. Its design prioritized inclusion: the path’s gentle gradients and broad layout enabled use by wheelchair users and patients transported in rolling chairs or carts, with dedicated staff in Meran available to assist as attendants—ensuring that therapeutic walking was not restricted by mobility limitations. It functioned as both medical infrastructure and a civic statement, embedding prevention into daily life rather than isolating it within institutions.

#### 5.3.2. Terrainkur and Modern Rehabilitation Science

The approach of Oertel anticipated core elements of modern exercise prescription, including workload stratification, dose–response logic, and outcome monitoring [74], principles that now underpin cardiac and pulmonary rehabilitation. Contemporary rehabilitation medicine has reaffirmed many of the principles embedded in the *Terrainkur* [77].

- Walking at low to moderate intensity yields significant benefits across cardiovascular, metabolic, and psychological domains, thereby affirming its pivotal role in preventive and rehabilitative medicine [78,79]. For patients with coronary artery disease, structured walking therapy—particularly when supervised—has been demonstrated to decrease cardiovascular mortality, hospitalizations, and myocardial infarction, and to result in measurable improvements in functional capacity and quality of life [80,81,82].
- In populations affected by obesity and metabolic syndrome, walking interventions have been shown to significantly enhance body mass index (BMI), insulin sensitivity, and inflammatory profiles.
- From a psychological perspective, walking is significantly correlated with reduced stress, anxiety, and depressive symptoms and enhances subjective well-being and quality of life. These effects are particularly pronounced in natural environments; “green” walking or park-based programs demonstrate greater improvements in mood and tension reduction than indoor or urban settings [83,84,85].

Table 1 summarizes the key health effects of low- to moderate-intensity walking across the cardiovascular, metabolic, and psychological domains. Its integration into structured public health strategies, including terrain-based therapies such as *Terrainkur*, anticipates and aligns with contemporary rehabilitation medicine. Tappeiner’s vision—encompassing tailored exertion, exposure to nature, and civic infrastructure as preventive medicine—is thus substantiated by a robust, contemporary evidence.

*Terrainkur* represents an early structural model of rehabilitation-oriented public health. Tappeiner’s contribution was not identifying walking’s health benefits, already intuitively recognized, but translating graded physical activity into a deliberately designed, publicly accessible environment.

This integration of dose-regulated movement, environmental exposure, and civic infrastructure anticipates contemporary rehabilitation and prevention frameworks: population-level physical activity promotion, walkability, and nature-based health strategies. The Tappeiner Promenade exemplifies health-oriented urban design, embedding rehabilitation principles into everyday space rather than institutional or individualized therapeutic settings.

### 5.4. Climate Medicine and Post-Tuberculosis Urban Health

Tappeiner’s initial endeavors to position Meran as a destination for individuals with respiratory conditions—grounded in meteorological analysis and longitudinal patient documentation [50]—foreshadowed contemporary methodologies that consider climate and topography as adjustable therapeutic resources. Currently, evidence supports the short-term effectiveness of low-altitude, clean-air environments in enhancing pulmonary function in patients with asthma and chronic obstructive pulmonary disease (COPD) [90]. Furthermore, prolonged exposure to variations in temperature, precipitation, and biometeorological conditions continues to be acknowledged as a significant determinant of chronic respiratory morbidity and exacerbations [91,92,93], thereby affirming Tappeiner’s assertion that Meran’s therapeutic efficacy was contingent upon climatic conditions and thus temporally constrained.

His subsequent transition towards terrain-based interventions, particularly through the establishment of the Tappeiner Promenade, signified a strategic reorientation of Meran’s role in health. As the town’s efficacy in treating infectious diseases diminished, Tappeiner reconceptualized it as a locus for recovery, prevention, and lifestyle-focused therapy, an approach that closely aligns with contemporary strategies for chronic disease prevention. In particular, the transition from passive climatic exposure to active, terrain-based physical activity marked a conceptual shift from environmental determinism to structured health behavior—a distinction of considerable relevance for contemporary physical activity promotion strategies. Modern public health increasingly prioritizes contextual behavior change, incorporating geography, environmental stimuli, and social infrastructure to design interventions that are both place-sensitive and scalable [94]. Nature-based and adventure therapies (NEATs), although not yet fully integrated into health systems, formally build upon this rationale by combining outdoor terrain with behavioral support to manage non-communicable diseases [95]. Techniques for behavior change, such as goal setting, monitoring, and knowledge shaping, are essential components of successful lifestyle interventions and are increasingly employed in low-resource settings [96,97].

**Figure 4:**
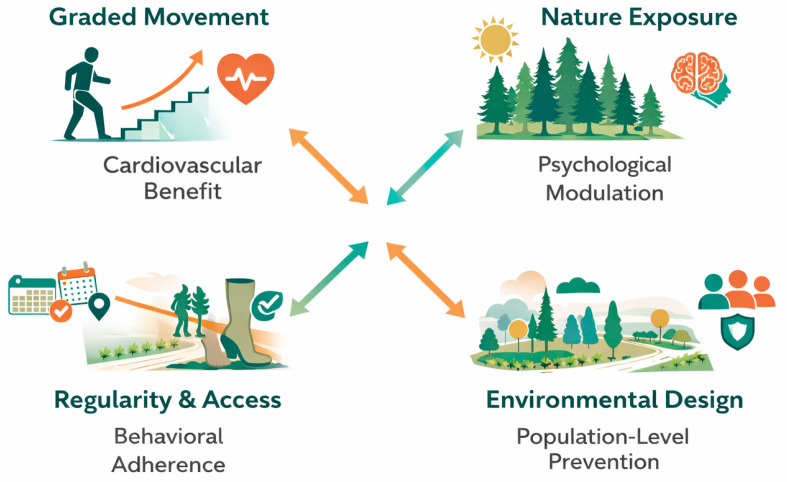
Mechanisms of terrain-based rehabilitation and prevention. This schematic illustrates the core therapeutic mechanisms underlying *Terrainkur*-based rehabilitation in contemporary research. It is a contemporary interpretative model inspired by Franz Tappeiner’s terrain-based health concepts and does not represent a historical illustration. Graded movement (**top left**) contributes to cardiovascular benefits through controlled physical exertion. Nature exposure (**top right**) modulates psychological well-being by reducing stress and enhancing mood. Regularity and access (**bottom left**) support behavioral adherence by embedding walking into daily routines using accessible infrastructure. Environmental design (**bottom right**) enables population-level prevention by integrating therapeutic landscapes into public-health planning. Colored arrows indicate synergistic interactions between domains, emphasizing the holistic and systems-based nature of *Terrainkur* as an individual and community health intervention.

In redefining Meran, Tappeiner shifted the focus from acute respiratory care to the management of chronic diseases through landscape-mediated approaches. This redefinition anticipates contemporary evidence-based spatial and behavioral strategies, as illustrated in Figure 4. His early vision recognized that both the environment and infrastructure serve not only as therapeutic contexts but also as active determinants of long-term health outcomes.

## 6. The Visionary: Integrating Clinical Insight, Environment, and Social Responsibility

A newspaper article published on Tappeiner’s 80th birthday in 1896 illustrates the extent to which his medical and civic work had entered public consciousness. The Meraner Zeitung portrayed him not merely as a successful physician, but as a moral authority and civic benefactor whose preventive, hygienic, and infrastructural initiatives were widely perceived as foundational for Meran’s transformation into a health resort [65]. This public framing underscores the societal resonance of preventive medicine and environmental health strategies in late-19th-century Meran.

Tappeiner’s trajectory—from tuberculosis care to terrain-based rehabilitation—embodied a synthesis of empirical medicine, environmental sensibility, and social equity. His efforts did not follow institutional blueprints; rather, they arose from situated practices, informed observations, and responsiveness to public needs.

In his writings and interventions, Tappeiner consistently argued for an expanded role for the physician—not limited to curative care, but extending into the design of spaces that facilitate physical activity, communication of health risks, and the democratization of prevention through accessible movement environments. He financed public infrastructure, distributed cholera pamphlets, documented meteorological-health correlations, and transformed his town into a health-promoting landscape.

Current public health frameworks increasingly endorse cross-sectoral collaboration, ecological determinants of health, and participatory civic engagement. In this respect, Tappeiner’s legacy extends well beyond mere historical curiosity. He emerges as a prescient model for integrating clinical practice, scientific reasoning, and civic ethics in public health. His work exemplifies what current scholarship calls the “clinician-citizen”—a physician whose responsibilities extend from bedside to policy, grounded in public health ethics and social accountability [98,99].

Tappeiner’s projects, such as advocating hygiene ordinances, supporting worker welfare, and investing in shared green spaces, directly anticipated today’s eco-social frameworks that view the environment, social equity, and health as interdependent systems [100,101]. His role in building the Tappeiner Promenade exemplifies this synthesis: a project shaped by environmental foresight, clinical insight, and civic commitment, realized through cooperation with local stakeholders. This project demonstrates that the promotion of physical activity in public health need not rely solely on individual counseling or educational campaigns but can be structurally embedded in the built environment—an insight of enduring relevance for contemporary health-promoting urban design. This aligns with modern calls for transdisciplinary, place-based health promotion, where municipalities, civil society, and health actors co-create public health infrastructure [102,103].

**Figure 5:**
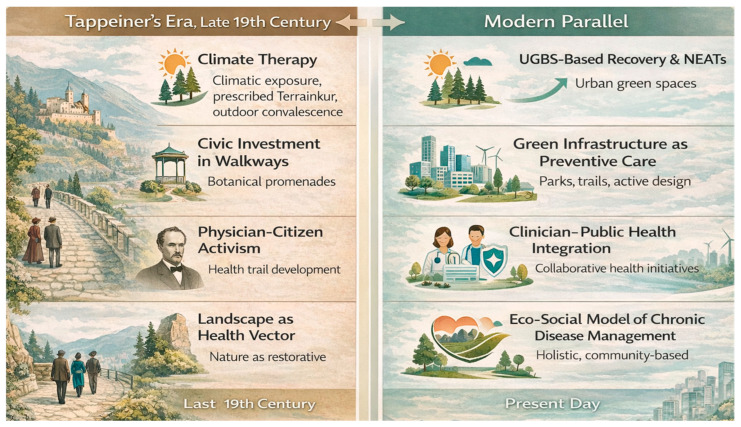
From Tappeiner’s Model to Modern Eco-Social Public Health. This two-panel schematic contrasts Tappeiner’s 19th-century health innovations with modern counterparts. Abbreviations: UGBS, Urban Green and Blue Spaces; NEATs, Nature-, Eco-, and Adventure-Based Therapies.

The Tappeiner Promenade—still in daily use—is not merely a historical artifact but a living embodiment of “health citizenship.” As a designed landscape that invites movement, reflection, and recovery, it resonates with current thinking around green citizen initiatives as health-promoting and place-making interventions [104]. It stands as a testament to how public health can be materially expressed as walkable terrain, civic space, and social infrastructure, all shaped by scientific care and public commitment (Figure 5).

## 7. Conclusions

Franz Tappeiner’s work shows how 19th-century medical practice extended beyond individual care to shape population health through environmental and civic intervention. This article reinterprets his activities through a public health lens: epidemic response, climate-based therapy, and terrain-based walking as early structured rehabilitation.

Tappeiner’s progression from cholera prevention and tuberculosis care to designed therapeutic walking infrastructure demonstrates continuity between historical *Terrainkur* and contemporary rehabilitation and prevention frameworks. His career illustrates how physical activity promotion can serve as a unifying principle across diverse public health domains—from infectious disease recovery to chronic disease prevention and mental health. His integration of graded physical activity, environmental exposure, and public accessibility anticipates walkability, green exercise, and health-promoting urban design.

The Tappeiner Promenade in Meran exemplifies this approach, embedding health promotion into everyday space rather than institutional settings. Given current challenges of physical inactivity, chronic disease and climate change, Tappeiner’s model demonstrates the enduring relevance of environmentally integrated, population-level strategies and the physician’s role as civic actor in shaping health-supportive environments that enable and sustain regular physical activity.

## Figures and Tables

**Table 1:** Health effects of low- to moderate-intensity walking across major health domains.

Health Domain	Target Population(s)	Observed Effects	Representative References
Cardiovascular	Inactive adults; coronary artery disease (CAD) patients	↓ Systolic/diastolic blood pressure, ↓ CV mortality, ↓ hospitalizations; ↑ VO_2_max, ↑ 6-min walk distance, ↑ quality of life	[78,80,82,86]
Metabolic	Obese adults and children; metabolic syndrome patients	↓ BMI, ↓ body fat, ↓ hs-CRP, IL-6, TNF-α; ↑ insulin sensitivity, improved HOMA-IR	[87,88,89]
Psychological & QoL	General population; chronic disease patients	↓ Stress, anxiety, fatigue, depression; ↑ mood, wellbeing, self-perceived health	[83,84,85]

The findings are based on randomized controlled trials and systematic reviews of both healthy and clinical populations. Arrows indicate the direction of effect: ↓ = decrease, ↑ = increase. Abbreviations: BMI, Body Mass Index; CV, Cardiovascular; CAD, Coronary Artery Disease; hs-CRP, High-Sensitivity C-Reactive Protein; IL-6, Interleukin-6; TNF-α, Tumor Necrosis Factor Alpha; HOMA-IR, Homeostatic Model Assessment of Insulin Resistance; VO_2_max, Maximal Oxygen Uptake; QoL, Quality of Life.

## Data Availability

No new data were created in this study.

## Supplementary Materials

The following supporting information can be downloaded at: https://www.mdpi.com/article/10.3390/ijerph23020248/s1, Figure S1: Original German Text of Franz Tappeiner’s 1855 Cholera Pamphlet; File S1: English Translation of Franz Tappeiner’s 1855 Cholera Pamphlet.

## Abbreviations

The following abbreviations are used in this manuscript:

BMI	Body Mass Index
COPD	Chronic Obstructive Pulmonary Disease
CV	Cardiovascular
HOMA-IR	Homeostatic Model Assessment of Insulin Resistance
NEATs	Nature-, Eco-, and Adventure-Based Therapies
QoL	Quality of Life
UGBS	Urban Green and Blue Spaces
VO_2_max	Maximal Oxygen Uptake
WASH	Water, Sanitation, and Hygiene
