# Supplementary material for: Walking for Health: Franz Tappeiner (1816–1902), Meran, and the Origins of Public Health-Oriented Physical Activity
Source: Int J Environ Res Public Health. 2026 Feb 16;23(2):248. doi: 10.3390/ijerph23020248 (PMC12941118; doi:10.3390/ijerph23020248)
Supplement: Supplementary file 1 [file ijerph-23-00248-s001.zip › ijerph-4128613-supplementary.pdf]

Supplementary Materials

Figure S1. Original German Text of Franz Tappeiner's 1855 Cholera Pamphlet. Image courtesy of the Palais Mamming Museum, Meran. © Palais Mamming Museum Meran.

Einige Worte über die Cholera

zur Belehrung und Beruhigung für das Landvolk.

Liebe Landsleute! Die Cholera steht wieder vor unserer Thür, und allenthalben verbreitet ihr Kommen Schrecken und Angst, wie vor 19 Jahren. „Warum?" frage ich. — „Weil Jedermann glaubt, daß wir noch ebenso rath- und hilflos der fürchterlichen Seuche gegenüberstehen, wie damals" — ist die allgemeine Antwort. Das ist aber ein Irrthum, ein schädlicher Irrthum; und es ist die Pflicht jedes Menschenfreundes, diesem Irrthume entgegenzutreten. Ja, liebe Landsleute! diese Meinung ist, Gott sei Dank, eine irrige. Die Medizin kennt die Cholera heute besser als damals. Sie hat zwar noch kein Mittel gefunden, die bereits vollkommen entwickelte Krankheit sicher zu heilen; aber sie hat entdeckt, daß *die Cholera nie plötzlich in voller Gestalt ausbricht, sondern daß sie immer einen Beginn, ein erstes Stadium hat, und was noch mehr ist, sie hat entdeckt, daß man die Krankheiit in diesem Beginne leicht und fast sicher heilen könne.* Das ist eine Thatsache, welche tausendfältige, gewissenhafte Erfahrungen bewährt und erprobt haben. Und eben in der unläugbaren Gewißheit dieser Thatsache liegt unser Trost, unsere Beruhigung. — Warum sollen wir uns auch vor der Cholera noch mehr fürchten, als vor jeder gewöhnlichen Krankheit? Es kommt ja nur darauf an, daß wir den Beginn der Cholera kennen, genau beachten und schnell das Mittel dagegen gebrauchen; dann sind wir der Heilung sicherer, als in jeder andern Krankheit. —

Dieser Beginn der Cholera ist immer eine kürzer oder länger dauernde Diarrhöe. Dieses „Abweichen" ist gewöhnlich leicht und schmerzlos, hat nur 2—4 halb oder ganz flüssige reichliche Stuhlentleerungen im Tage zur Folge, stört das gesunde Befinden meistens gar nicht, oder ist höchstens mit etwas Mattigkeit und Verdauungsbeschwerden verbunden. Dieses Stadium dauert manchmal mehrere Tage, häufig aber einen oder einen halben Tag, bisweilen nur einige Stunden; dann erst bricht die Cholera in voller, schrecklicher Gestalt aus. Aber eben, weil dieses Stadium so leicht und unscheinbar ist, wurde es leider bisher meistens übersehen.

Diesen Beginn nun, diesen ersten Durchfall müßt Ihr sorgsam beachten, lieben Landsleute. Man braucht dazu kein besonderes Studium. Jeder merkt es leicht, wenn er Acht giebt, und sobald er es bemerkt, so säume er nicht, schnell das sichere Mittel dagegen anzuwenden. Dies Mittel ist einfach und wohlfeil, es besteht in folgenden Pillen:

Recept. Opii puri gran. decem.
Pulv. Catechu gran. viginti.
Extr. chamomill. q. s. f. l. a pill.
No. viginti. Consp. Liq. Dt.

Von diesen Pillen nimmt man alle 3 Stunden 1 Stück, bei stärkerer Diarrhöe alle 2 Stunden, selbst alle Stunden 1 Stück, esse dabei nichts als eingebrennte Fleisch- oder Wassersuppe, trinke nur Wasser und kleide sich warm, und fahre damit fort, bis der Durchgang wieder in Ordnung ist. Bei Kindern zerdrückt man die Pillen und giebt ihnen je nach ihrem Alter jedesmal 1/8 — 1/4 von einer Pille. — Natürlich muß, so lange die Cholera an einem Orte herrscht, dasselbe Verfahren wiederholt werden, so oft sich wieder die Diarrhöe einstellt. —

Eine solche Dosis Pillen kostet nur 13 kr. Es kann daher und soll sich Jedermann selbe im Voraus anschaffen. Den Armen werden sie edle Menschenfreunde umsonst besorgen.

Denn an der Hand muß man das Mittel haben, wenn man seiner Sache sicher sein will; man soll es immer bei sich führen. Niemand weiß, wo und wann ihn der Beginn der Cholera packt — auf dem Felde, auf der Reise, im Bette 2c., und Niemand weiß auch, wie lange bei ihm dies Stadium dauert, und *nur in diesem Stadium hilft das Mittel.* Muß er erst stundenlang auf den Arzt und die Medizin warten, so vergeht die beste Zeit, die Cholera bricht unterdessen aus, und das Leben ist in größter Gefahr.

Und sollte auch der Fall eintreten, liebe Landsleute, was Gott geben wolle, daß Ihr die Pillen für die Cholera nicht braucht, so sind sie deßwegen nicht unnütz, Ihr könnt sie bei jedem andern Durchfall und Grimmen mit gutem Erfolge gebrauchen. —

Zum Schlusse noch ein paar Worte über die Art und Weise, wie man zur Cholerazeit leben soll. Manche zittern fast bei jeder Speise und getrauen sich das Zehnte nicht zu essen. Wieder Andere rathen, recht toll und voll zu essen und zu trinken. Die Wahrheit liegt auch hier in der Mitte. Alles Uebermaaß ist schädlich. Jeder bleibe bei seiner gewohnten Lebensweise, welche er erfahrungsmäßig am besten verträgt, gehe seine Wege und Stege, kleide sich allenfalls etwas wärmer als gewöhnlich, vermeide Verkühlungen und Durchnässungen und ziehe in letzterem Falle sobald als möglich trockene Wäsche an, trinke nicht kalt bei erhitztem Körper, und enthalte sich vor unreifem Obst, Gurken, Pflaumen und Salat und schlechtem Wein und Bier. Reinlichkeit und Lüftung der Schlafzimmer, aber auch gehöriger Verschluß derselben zur Nachtzeit ist sehr zu empfehlen, so wie Maaß und Ordnung in Allem, selbst in der Arbeit.

Ich füge noch zur Beruhigung bei, daß die Cholera im Durchschnitt nicht ansteckend ist, daher auch Aerzte und Geistliche selten davon befallen werden. Der Cholera-Stoff verbreitet sich durch die Luft, von der sich Niemand absperren kann. Man darf sich daher nicht fürchten, seinem leidenden Mitbruder auf jede Art beizustehen.

Gott verleihe meinen Worten Kraft und Vertrauen und gebe seinen Segen dazu! —

Meran, den 1. August 1855.

Dr. Tappeiner.

Eberle'sche Buchdruckerei in Bozen.

File S1. English Translation of Franz Tappeiner's 1855 Cholera Pamphlet.

A few words about cholera
for the education and reassurance of the rural population.

Dear fellow countrymen! Cholera is once again at our doorstep, and its arrival is spreading terror and fear everywhere, just as it did 19 years ago. "Why?" I ask. "Because everyone believes that we are still as helpless and powerless in the face of this terrible epidemic as we were then" — is the general answer. But this is a mistake, a harmful mistake; and it is the duty of every humanitarian to counteract this mistake. Yes, dear compatriots! Thank God, this opinion is mistaken. Medicine knows more about cholera today than it did then. It has not yet found a cure for the fully developed disease, but it has discovered that cholera never breaks out suddenly in its full form, but always has a beginning, an initial stage, and what is more, it has discovered that the disease can be easily and almost certainly cured in this initial stage. This is a fact that has been proven and tested by thousands of conscientious experiments. And it is precisely in the undeniable certainty of this fact that we find our comfort and reassurance. Why should we fear cholera any more than any other common disease? All that matters is that we recognise the onset of cholera, observe it closely and quickly administer the remedy; then we are more certain of a cure than with any other disease.

The onset of cholera is always characterised by diarrhoea of varying duration. This "deviation" is usually mild and painless, resulting in only 2-4 semi-liquid or completely liquid bowel movements per day, and usually does not interfere with health at all, or is associated with at most a slight feeling of fatigue and indigestion. This stage sometimes lasts several days, but often only a day or half a day, sometimes only a few hours; only then does cholera break out in all its terrible glory. But precisely because this stage is so mild and inconspicuous, it has unfortunately been overlooked in most cases until now.

You must pay close attention to this beginning, this first bout of diarrhoea, dear compatriots. No special study is required. Anyone can easily notice it if they pay attention, and as soon as they notice it, they should not delay in quickly applying the sure remedy. This remedy is simple and inexpensive, consisting of the following pills.

Recipe. **Opii puri gran. Decem.**
Pulv. Catechu gran. viginti.
Extract of chamomile q.s. for one pill.
No. Viginti. Consp. Liq. Dt.

Take one of these pills every three hours, or every two hours in cases of severe diarrhoea, or even one every hour, eating nothing but boiled meat or water soup, drinking only water and dressing warmly, and continue this until your bowel movements return to normal. For children, crush the pills and give them ⅛ — ¼ of a pill at a time, depending on their age. Of course, as long as cholera is prevalent in a place, the same procedure must be repeated as often as diarrhoea recurs.

Such a dose costs only 13 kr. Therefore, everyone can and should purchase it in advance. Kind-hearted people will provide it free of charge to the poor.

For you must have the remedy at hand if you want to be sure of your cause; you should always carry it with you. No one knows where and when cholera will strike — in the field, while travelling, in bed, etc. — and no one knows how long this stage will last, and the remedy only helps at this stage. If you have to wait hours for the doctor and the medicine, the best time will pass, cholera will break out in the meantime, and your life will be in grave danger.

And even if, dear compatriots, God forbid, you do not need the pills for cholera, they are not useless. You can use them with good results for any other diarrhoea or stomach ache.

Finally, a few words about how to live during cholera season. Some people tremble at almost every meal and do not dare to eat a tenth of what they should. Others advise eating and drinking heartily

and to excess. The truth lies somewhere in the middle. All excess is harmful. Everyone should stick to their usual lifestyle, which they know from experience suits them best, go about their business, dress a little warmer than usual if necessary, avoid getting cold and wet, and in the latter case, change into dry clothes as soon as possible, do not drink cold drinks when your body is hot, and refrain from eating unripe fruit, cucumbers, plums, salad, bad wine and beer. Cleanliness and ventilation of bedrooms, but also proper closure of the same at night, is highly recommended, as is moderation and order in everything, even at work.

I would like to add, for your reassurance, that cholera is not contagious in general, which is why doctors and clergy are rarely affected by it. The cholera material spreads through the air, from which no one can shield themselves. Therefore, one should not be afraid to assist one's suffering fellow man in any way.

May God give my words strength and confidence and bless them! —

Merano, 1 August 1855.

Dr. Tappeiner

Eberle Printing House in Bolzano.
